# The hyperdense basilar artery sign: a case of locked-in syndrome

**DOI:** 10.1186/s12245-016-0104-9

**Published:** 2016-02-29

**Authors:** Lasanthi Aryasinghe, Yasmin Kazim, Hamza F. Obeid, Husnain Hashim

**Affiliations:** Department of Emergency Medicine, Rashid Hospital Trauma Center, P.O. Box 53378, Dubai, UAE; Department of Internal Medicine, Rashid Hospital Trauma Center, Dubai, United Arab Emirates; Department of Neurology, Rashid Hospital Trauma Center, Dubai, United Arab Emirates

**Keywords:** Locked-in syndrome, Basilar artery thrombosis, Basilar artery occlusion, Posterior circulation stroke, Hyperdense basilar artery sign

## Abstract

**Background:**

Locked-in syndrome, although a notoriously famous clinical entity, the rarity of the condition coupled with the variability of clinical features on acute presentation represents a potential diagnostic pitfall for the emergency physician.

**Case:**

A previously healthy 25-year-old female was brought to our Emergency Department after being found unresponsive. On examination, she was conscious and alert with a Glasgow Coma Score of 9; on neurological examination, the patient was quadriplegic and unable to speak but was able to move her eyes and blink. Non-contrast brain computed tomography (CT) revealed a hyperdense basilar artery, and CT cerebral angiography confirmed basilar artery thrombosis.

**Conclusion:**

This case highlights the need for a high index of suspicion to make a diagnosis of locked-in syndrome in the Emergency Department, especially in young patients with no apparent risk factors for an ischemic stroke. The hyperdense basilar artery sign is one of the earliest signs on non-contrast CT imaging and may be the only clue to guide further management in a patient with basilar artery occlusion.

## Case presentation

A previously healthy 25-year-old female was brought to the Emergency Department after being found frothing at the mouth, unresponsive, and stiff all over. The patient had been complaining of headache, neck and shoulder pain for 2 days prior and was prescribed ibuprofen, no other history was available.

On examination, the patient was conscious and alert but appeared extremely distressed, continuously frothing at the mouth with trismus and tongue protrusion requiring regular suctioning; however, she was able to maintain her airway and all vital signs were within normal limits. She was also tearing and groaning frequently.

On neurological examination, the patient’s initial Glasgow Coma Score was 9 (eyes - 4, verbal - 2, motor - 3), her head was lateralized to the right, she was able to move her eyes and blink to command but was unable to speak, pupils were 3 mm equal and reactive, and her right upper limb was stiff and flexed (unilateral decorticate rigidity) with no movement of her left side even to noxious stimulus. Motor exam revealed increased tone with hyperreflexia in all extremities and bilateral extensor plantar reflex (upgoing plantars).

**Fig. 1:**
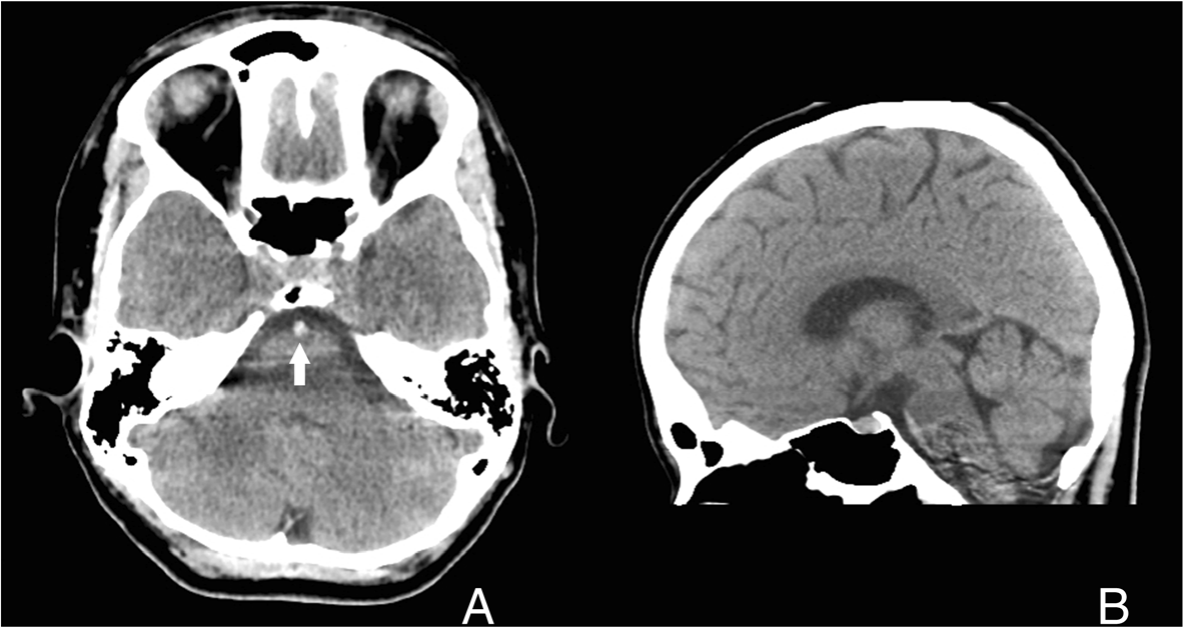
**a** Non-contrast CT (*axial view*) showing the hyperdense basilar artery sign (*arrow*). **b** Non-contrast CT (*sagittal view*) with the hyperdense basilar artery seen anterior to the pons

**Fig. 2:**
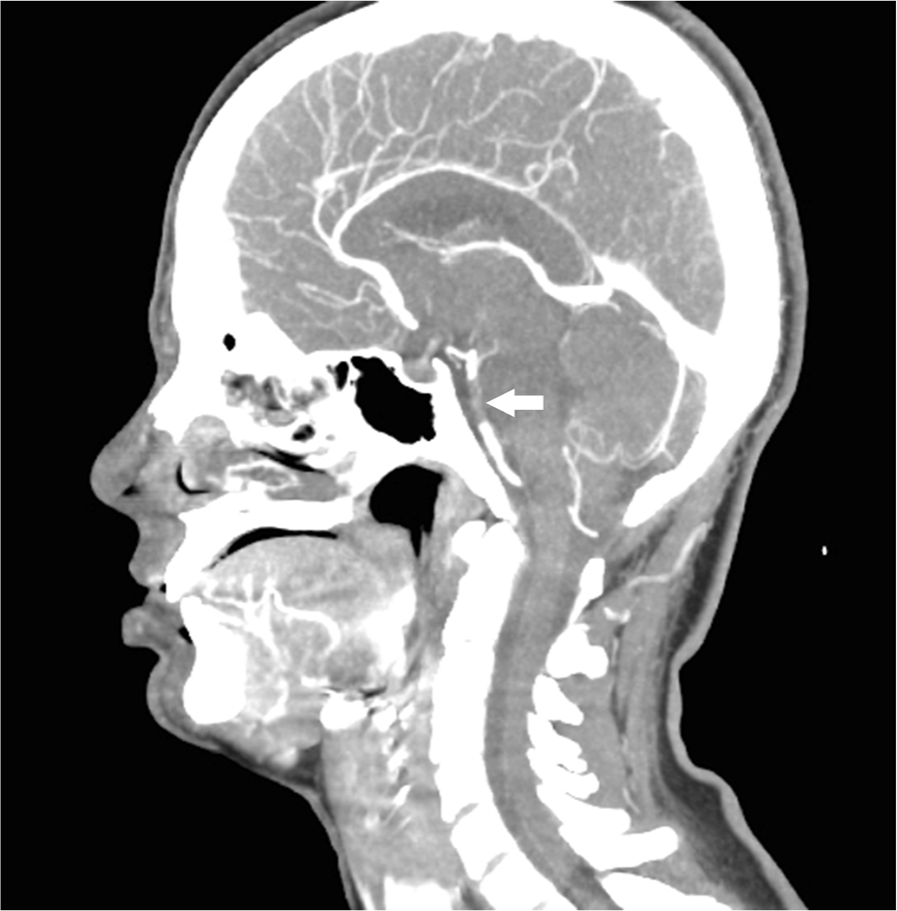
CT angiography (*sagittal view*) revealed a 1-cm filling defect of the basilar artery (*arrow*)

Non-enhanced computed tomography of the brain revealed a hyperdense basilar artery (Fig. 1a, b) suggestive of basilar artery thrombosis, and CT cerebral angiography confirmed a 1-cm occlusion of the basilar artery (Fig. 2).

Our patient was deemed not a candidate for thrombolysis and was managed conservatively due to several factors including a late presentation (over 10 h from last known normal) and remained locked-in at the time of her discharge from hospital.

## Background

Locked-in syndrome (LIS) is a devastating clinical condition, the rarity of the condition coupled with the variability of clinical features on acute presentation represent a potential diagnostic pitfall for the emergency physician. It typically occurs as a result of an ischemic insult to the ventral pons due to thrombosis of the basilar artery but may also be seen due to hemorrhage, vertebrobasilar dissection, tumors, central pontine myelinolysis, or even multiple sclerosis involving the ventral pons [1].

Occlusion of the basilar artery, being the main vessel of the posterior circulation may produce nonspecific symptoms ranging from headache or vertigo to isolated cranial nerve palsies, hemiplegia, locked-in syndrome, or even coma [2]. Diagnosis of LIS hinges on the clinical prerequisites of quadriplegia or quadriparesis and the inability to speak (anarthria) with preservation of consciousness and eye movements or blinking. Three categories of LIS have been described; the *classic* in which the patient has intact vertical eye movements, *incomplete LIS* which is similar to the classic with remnants of some voluntary motor control, and *total LIS* in which the patient is fully conscious but has complete immobility including eye movements [1].

As with any acute stroke, a non-enhanced head CT should be performed promptly; the hyperdense basilar artery sign (Fig. 1a), similar to the hyperdense middle cerebral artery sign in anterior circulation strokes, may be the only finding in an acute presentation and has been shown to be a strong predictor of basilar artery thrombosis in patients with a high pretest probability for a posterior circulation stroke (71 % sensitivity, 98 % specificity) [3, 4].

Emergency Department management of these patients involve airway and hemodynamic management, with neuromedical consultation for intravenous thrombolysis within 4.5 h of symptom onset or intra-arterial thrombolysis (IAT) within 6 h [2]; with some studies advocating the use of IAT in acute basilar artery occlusions 12 to 24 h after symptom onset [5] due to the extremely poor functional prognosis of the condition. In patients with failed recanalization after IV thrombolysis, studies have shown promising results with the use of mechanical endovascular interventions [6].

## Conclusions

Awareness of the clinical features suggestive of a posterior circulation stroke and recognition of the hyperdense basilar artery sign on non-contrast CT, with rapid confirmation of the diagnosis via multimodal CT or MRI and initiation of intravenous or intra-arterial thrombolysis will decrease the high mortality and morbidity associated with this devastating condition.

## Consent

Written informed consent was obtained from the patient’s next of kin for publication of this report and accompanying images. A copy of the written consent is available for review by the Editor-in-Chief of this journal on request.
